# Monitoring the regulation of gene expression in a growing organ using a fluid mechanics formalism

**DOI:** 10.1186/1741-7007-8-18

**Published:** 2010-03-04

**Authors:** Rémy Merret, Bruno Moulia, Irène Hummel, David Cohen, Erwin Dreyer, Marie-Béatrice Bogeat-Triboulot

**Affiliations:** 1 INRA, Nancy Université, UMR1137 Ecologie et Ecophysiologie Forestières, IFR 110 EFABA, F-54280 Champenoux, France; 2 INRA, Université Blaise Pascal, UMR_A547 Physique et Physiologie Intégratives de l'Arbre Fruitier et Forestier, Domaine de Crouelle, F-63039 Clermont Ferrand, France

## Abstract

**Background:**

Technological advances have enabled the accurate quantification of gene expression, even within single cell types. While transcriptome analyses are routinely performed, most experimental designs only provide snapshots of gene expression. Molecular mechanisms underlying cell fate or positional signalling have been revealed through these discontinuous datasets. However, in developing multicellular structures, temporal and spatial cues, known to directly influence transcriptional networks, get entangled as the cells are displaced and expand. Access to an unbiased view of the spatiotemporal regulation of gene expression occurring during development requires a specific framework that properly quantifies the rate of change of a property in a moving and expanding element, such as a cell or an organ segment.

**Results:**

We show how the rate of change in gene expression can be quantified by combining kinematics and real-time polymerase chain reaction data in a mechanistic model which considers any organ as a continuum. This framework was applied in order to assess the developmental regulation of the two reference genes *Actin11 *and *Elongation Factor 1-β *in the apex of poplar root. The growth field was determined by time-lapse photography and transcript density was obtained at high spatial resolution. The net accumulation rates of the transcripts of the two genes were found to display highly contrasted developmental profiles. *Actin11 *showed pulses of up and down regulation in the accelerating and decelerating parts of the growth zone while the dynamic of *EF1β *were much slower. This framework provides key information about gene regulation in a developing organ, such as the location, the duration and the intensity of gene induction/repression.

**Conclusions:**

We demonstrated that gene expression patterns can be monitored using the continuity equation without using mutants or reporter constructions. Given the rise of imaging technologies, this framework in our view opens a new way to dissect the molecular basis of growth regulation, even in non-model species or complex structures.

## Background

Understanding time-course changes in gene expression and their complex interplay is a major challenge in gene regulation networks [1,2]. Attention has been shifted from mRNA steady-state levels towards rates of changes in transcript abundance and their regulation [3-5]. These dynamic of gene expression become even more complex when dealing with the developmental biology of multicellular bodies. Indeed, the fact that both temporal and positional cues are known to directly influence transcriptional networks [6] creates the need for a spatiotemporal specification of the changes in gene expression. Coordinated gene expression in tissue (or cells) at different stages of development has been revealed using technologies such as microarray analyses [7,8], mutant screening [9,10] and novel imaging techniques [11,12]. However, these approaches only provide snapshots of gene expression at given times or developmental stages. In these widespread cases, gene expression is mapped in an Eulerian specification, using spatial coordinates at a given time. In developing multicellular structures, changes in gene expression occur alongside changes in organ size, shape and anatomy. Cell positions thus vary continuously due to cell movements, cell growth and cell death [13,14]. It is thus relevant to follow a cell during its movement over time in the growing tissue to assess gene expression along the developmental trajectory through a material or Lagrangian specification. Despite evidence of important gene regulations following cell movements [15], there has so far been little research into quantitatively monitoring the rates of changes in expression levels over time and position during the developmental movements.

The question of how cell expansion and cell displacement both influence organ growth and gene expression can be addressed using mechanistic models that consider an organ as a continuum [13,15]. Quantifying rates of change in moving elements has been a longstanding issue in continuum mechanics. This approach requires a detailed characterization of the motion velocity field v(x, t) which can be monitored using video or time-lapse photography [15,16]. Based on the conservation of mass in a deforming continuum, the continuity equation properly quantifies the rate of change of a property in a moving element [17]. For instance, if the concentration of compound A varies across a growing tissue but not with time (that is, A(t) is steady but A(x) is not constant), then the local rate of change ∂A/∂t at any position x is zero. However, there has necessarily been a change in the concentration of compound A in the moving element to match the change between A(x) and A(x+v.dt) (convective changes). By the same token, if the element volume has increased between t and t+dt, then a constant A over time means that net synthesis has occurred and compensated the dilution due to volumetric growth. The continuity equation is able to take into account not only the local rate of change but also the consequences of movement and of possible changes in volume on the rate of change of the property [18].

The use of the continuity equation and the underlying kinematic analyses for growth studies was developed in the 1950's [19-21]. It was then introduced to a wider audience [22,23] and reviewed in [18,24]. Plant tissues can easily be considered as a continuum: since plant cells are stuck together by their middle lamella, shear between cells is usually infinitesimal. Consequently, cell motion in a growing organ is due to the elongation of other cells pushing it [24]. To date, the continuity equation has mainly been applied to gain insights into the physiology of both growth and development of elongating organs. For example, the continuity equation framework has revealed overwhelming convective components of the rate of change of uronide deposition in growing roots [25]. It has also been used to assess the effects of stresses on growth [26,27].

Surprisingly, this framework has not yet been applied to gain a spatiotemporal description of the rates of change in gene expression and provide molecular developmental information in growing tissue. In the present work, changes in the rate of gene expression in the apex of poplar roots growing under optimal conditions were quantified at high spatial resolution with real-time polymerase chain reaction (real-time PCR). We assessed the developmental regulation of two genes: *Actin11 *and *Elongation Factor 1-β*. These two genes are widely used as internal controls in real-time PCR [28]; yet both may be under transcriptional regulation in time and/or space in an actively growing organ. We show that the rate of change in gene expression can be quantified in time and space by combining kinematics and the continuity equation with real-time PCR. The resulting fields of expression rate revealed highly contrasted profiles for the control of *Actin11 *and *Elongation Factor 1-β *during the developmental movement. We highlighted the importance of the convective component for monitoring changes in gene expression. These results underline that careful attention must be paid to growth and its possible alteration induced by changes in the environment before drawing any conclusions from raw transcript profiling in growing organs [29]. We demonstrate that the regulation of gene expression can be evidenced in growing tissues without using mutants or reporter constructions. Consequently, the proposed framework also constitutes in our view a useful bypass for in-depth analysis of the molecular basis of growth regulation in a wide range of non-model species.

## Results

### Trajectory, velocity and relative elemental growth rate along the growth zone

**Figure 1:**
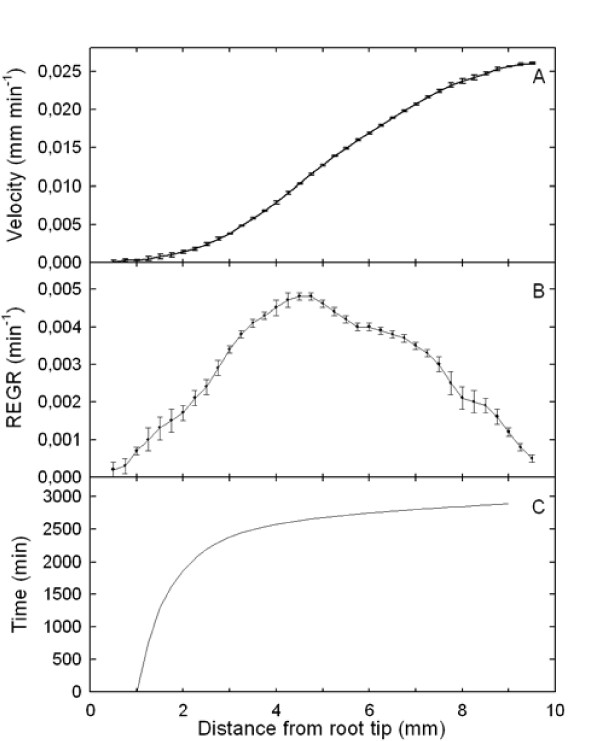
**Velocity, relative elemental growth rate and growth trajectory along the root apex**. (A) Displacement velocity profile along the root apex (reference point is the root tip). Mean ± standard error of mean (SEM; *n *= 6). (B) Relative elemental growth rate (spatial derivative of displacement velocity) along the root apex. Mean ± SEM (*n *= 6). C: Growth trajectory of a meristem-derived element initially located at 1 mm from the root tip - the time at which it reached a given distance from the tip along its developmental movement away from the meristem. Growth trajectory was calculated from mean velocity data. It was drawn only from 1 mm onwards, since velocity was almost nil in the apical first mm, making the calculated displacement time uncertain.

The growth-velocity profile (velocity of cell movement away from the root tip) was determined in 6 roots aged from 5 to 11 days (Figure 1A). As shown by the very low variability around the average profile, neither root age nor the time of the observation along the night (from 1 h to 6 h after light shutting, see Additional File 1: Figure S1A) induced significant changes in the growth profiles. A side experiment comparing root growth during the light phase and the night phase did not reveal any change due to direct light signaling or possible nycthemeral rhythm (see Additional File 1: Figure S1B). Growth was thus assumed to be steady for a few days in the following. The spatial derivative of velocity - that is, the relative elemental growth rate measuring relative elongation per unit time of elements along the axis, had a skewed bell-shaped distribution peaking at 4.5 mm from the root tip (Figure 1B). The first 2 mm, which include the root cap and the meristematic zone, displayed low velocity. Velocity increased with distance from the root tip, reaching a plateau of 0.026 mm min^-1 ^at approximately 9 mm, corresponding to the elongation rate of the whole root. Given the assumption of steady state, the growth trajectory - that is, the mapped position on the root of a meristem-derived tissue element as a function of time was calculated from velocity data. It was found highly non-linear (Figure 1C). A tissue element crossed the growth zone in about 2800 min (about 2 days), but spent more than 2300 min (over 38 h) in the 1-3 mm section - over 80% of total growth duration.

### Transcript density along the growth zone

**Figure 2:**
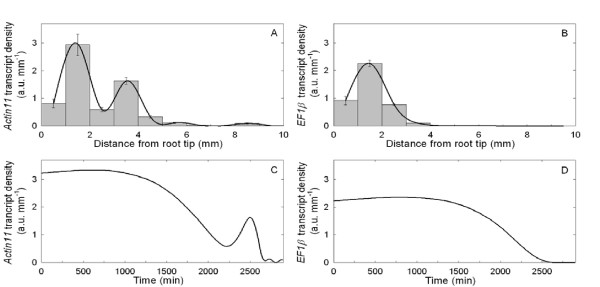
**Spatial and temporal specifications of *Actin11 *and *EF1β *transcript density**. *Actin11 *(A) and *EF1β *(B) transcript density (a.u. mm^-1^) in 1 mm-long segments of the primary root. Mean ± standard error of mean (SEM; *n *= 3). The black line corresponds to the cubic spline interpolation of the means. *Actin11 *(C) and *EF1β *(D) transcript density as a function of time in an element moving across the growth zone. Mean ± SEM (*n *= 3).

As the growth zone shows highly heterogeneous local velocity and relative elemental growth rate (Figure 1A and 1B), the spatial distribution of molecular entities was analysed at high spatial resolution. The transcript densities of *Actin11 *and a putative *Elongation Factor 1-β *(*EF1β*) were plotted against the current segment position on the root axis (Eulerian specification, Figure 2A and 2B). The *Actin11 *density profile displayed two peaks at 1.5 and 3.5 mm from the root tip with similar spans, but decreasing intensity, and low values in more distal segments. The *EF1β *density profile differed from that of *Actin11*, displaying a single peak at 1.5 mm and very low levels in all the more distal segments.

No time trend of the RNA profiles can be suspected (see Additional File 2: Figure S2A), suggesting a temporal stability of the RNA profiles whatever the age of the root, or the time of the observation during the night phase. Consistently with the steadiness of growth, the profiles of transcript density were thus assumed to be steady for a few days in the following (see also Additional File 2: Figure S2B for additional indirect arguments). Assuming steady growth and steady transcript density, the developmental time-course of the transcript density in a tissue element initially positioned at 1 mm from the root tip (Lagrangian specification, Figure 2C and 2D for *Actin11 *and *EF1β*, respectively) was calculated by combining transcript density (Figure 2A and 2B) with growth trajectory (Figure 1C). Due to non-linear changes in local velocity, the actual time-course of transcript density in a given tissue element was quite different from what could be expected intuitively from Figures 2A and 2B. For *Actin11 *(Figure 2C), the first peak lasted almost five times longer than the second peak, indicating that the first changes in *Actin11 *were smooth and long-lasting, whereas the second peak was a transient shift (8 h). However, even these transcript density profiles (Figure 2) cannot be analysed for regulation of gene expression simply by considering position-to-position variations, since as time goes on, each element not only moves but also expands (Figure 1B). While the Lagrangian specification of transcript density considers the movement of the tissue element, only the continuity equation gives an unbiased view of gene expression during growth, taking into account both cell movement and cell volume expansion.

### Regulation of gene expression

**Figure 3:**
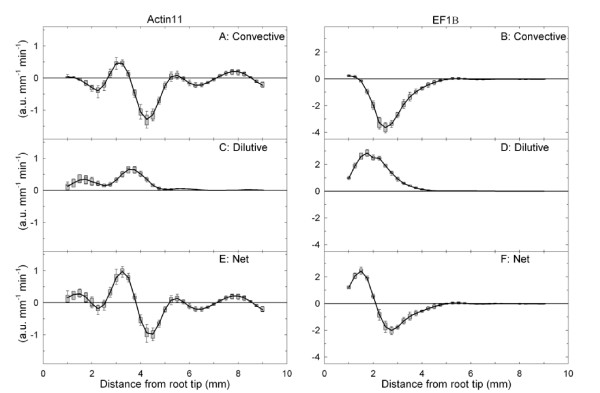
**Material derivative of *Actin11 *and *EF1β *transcript densities**. Convective (A, B) and dilutive (C, D) components of the material derivative (E, F) - the net accumulation rate of *Actin11 *and *EF1β *transcripts, respectively - as a function of the distance from the root tip (Eulerian specification). The material derivative and its components are expressed in arbitrary unit mm^-1 ^min^-1^. The black lines were calculated from the mean values of each parameter using equation (3). Box-and-whisker plots are the distribution of 1000 replicates (obtained by random resampling). The central mark is the median, the edges are the 25th and 75th percentiles, and the whiskers extend to 10th and the 90th percentiles.

The material derivative of transcript density D (expressed in arbitrary unit mm^-1 ^min^-1^) was calculated from the continuity equation. D corresponds to the net accumulation rate of transcripts in each element along the root. In other words, D reveals the regulation of gene expression resulting from the balance of transcription and decay along the root, corrected for the positional and dilutive effects of growth (if D = 0, then transcript level remains constant). From equation (3), it is possible to plot the two components of D, namely the convective and the dilutive components. Under the assumption of steady transcript density, the local rate of change in gene expression δρ/δt is null. The convective rate of change, v.δρ/δx, corresponds to the variation in transcript density in an element moving to a region further away from the root tip (Figure 3A and 3B). The dilutive component, ρ.δv/δx (Figure 3C and 3D) assesses potential compensation of the dilution of transcript abundance during expansive growth through net transcript synthesis (that is, if ρ.δv/δx = 0, then net transcript synthesis just compensates for the expansion-driven dilution).

Figures 3E and 3F illustrate these net accumulation rates along the root growth zone (Eulerian specification) for the *Actin11 *and *EF1β *transcripts, respectively. For both genes, the profile of the net accumulation rate of transcripts in tissue elements D was highly dynamic in the zone of increasing relative elemental growth rate (Figure 1B). In the distal part of the growth zone (5-9 mm), D was generally not significantly different from zero, indicating that the transcript level remained almost constant for both genes. There was an active accumulation of *Actin11 *transcripts peaking at 3.25 mm in cells undergoing growth acceleration, followed by a negative peak of almost the same amplitude at 4.25 mm (Figure 3E) in cells just starting to decelerate their growth (Figure 1B). Significant peaks of smaller amplitude were observed, showing that *Actin11 *transcripts accumulate in cells at 1.5 mm from the root tip (probably sitting in the division zone) and in cells that were poised to enter post-growth cell differentiation at 8.5 mm. In comparison, the *EF1β *expression is up-regulated closer to the root tip, at 1.5 mm (Figure 3F). As for *Actin11*, this accumulation phase was followed by a phase during which the decay rate overcame the transcription rate. For *Actin11*, the absolute value of D was clearly mainly attributable to the convective component whereas for *EF1β *the relative contribution of the convective and dilutive components was balanced (Figure 3), meaning that synthesis rates were unequal for both transcripts. Note also that net transcript synthesis overreached dilution in the first 5 mm - the zone of increasing relative elemental growth rate (Figure 3C and 3D). Beyond 5 mm, the dilutive effect was just compensated.

**Figure 4:**
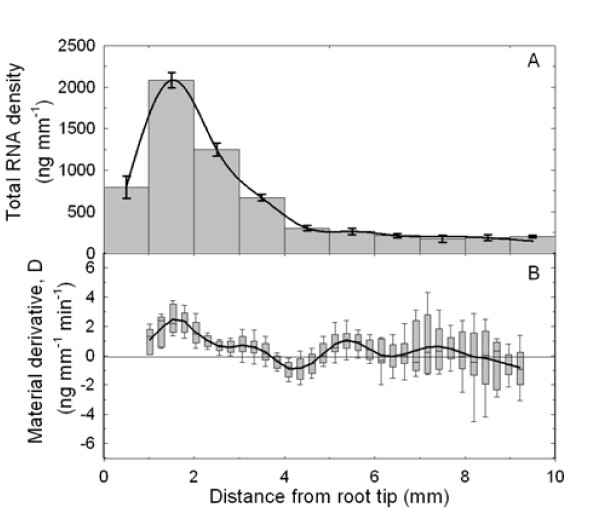
**Total RNA density and its material derivative**. (A) Total RNA density (ng mm^-1^) in 1 mm-long segments of the primary root. Mean ± standard error of mean (*n *= 3). The black line corresponds to the cubic spline interpolation of the means. (B) Material derivative - the net accumulation rate, of total RNA (ng mm^-1 ^min^-1^). The black line was calculated from the mean values of each parameter using equation (3). Box-and-whisker plots are the distribution of 1000 replicates (obtained by random resampling). The central mark is the median, the edges are the 25th and 75th percentiles, and the whiskers extend to 10th and the 90th percentiles.

Since the density of nuclei and presumably the cellular activity are heterogeneous along the root growth zone [29,30], the net accumulation rate of total RNA was used as a control. The total RNA content varied significantly along the growth zone, with a lognormal distribution and a peak at 1.5 mm from the tip (Figure 4A). The net accumulation rate of total RNA, calculated using the same formalism as above, was positive in the 1-3 mm range and showed a flat and near null profile thereafter (Figure 4B). The simulated distribution of D, shown by box-and-whiskers plots, included zero, meaning that the mean value of D can be null by chance. In other words, the net accumulation rate of total RNA can not be considered as strictly different from zero beyond the 3 mm mark (Figure 4B). This location of total RNA accumulation adds to the evidence for a high cellular activity of a meristematic zone.

**Figure 5:**
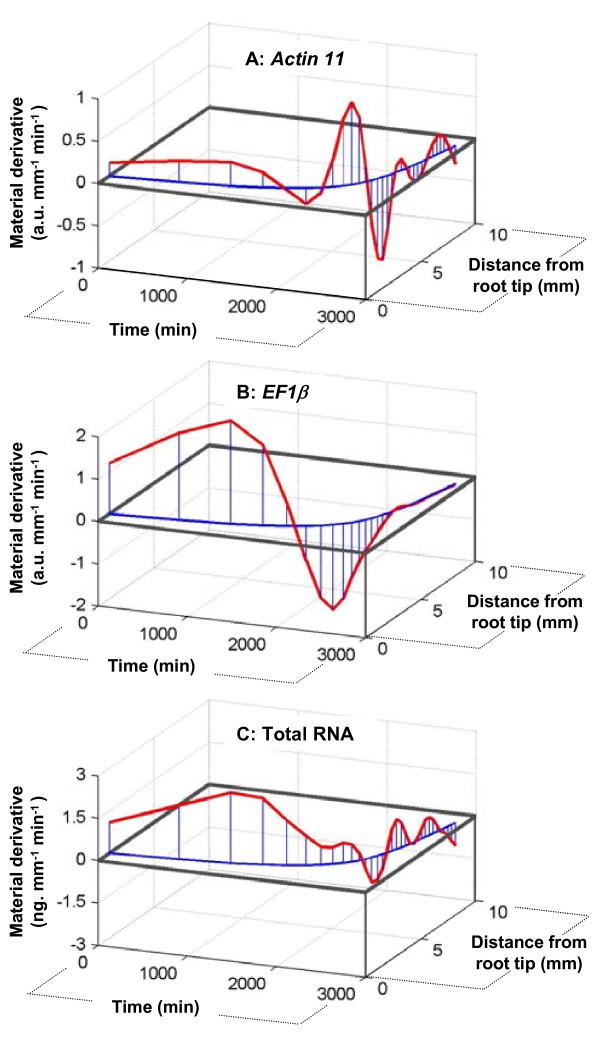
**Spatio-temporal mapping of gene regulation**. Lagrangian representation of *Actin11 *(A), *EF1β *(B) transcript accumulation rate (a.u. mm^-1 ^min^-1^) and total RNA accumulation rate (C) (ng mm^-1 ^min^-1^) along the primary root apex and with time. Net accumulations rates (Z axis) are plotted along the growth trajectory of an element (curve in the XY, see Figure 1C). Computed on Matlab software.

Given the calculation of temporal variations using the growth trajectory (as in Figure 2C and 2D), net accumulation rates were plotted in space and time (Figure 5, Additional Files 3, 4, 5: Movie1, Movie2 and Movie3). In other words, variations of net accumulation rate were tracked in a tissue element which was originally located at 1 mm from the root tip and moved through the growth zone away from the meristem. This spatiotemporal mapping enables to equally perceive kinetic and positional cues. The Lagrangian specification is shown here for a single element, but as long as growth and development are steady, all the elements display the same developmental fate [18]. Figure 5 highlights that the variations in net accumulation rate of *Actin11 *and *EF1β *transcripts differed markedly from the dynamics of total RNA, thus strengthening the conclusion of a tight regulation of gene expression in any cell crossing the growth zone. Given that total RNA accumulation rate did not significantly differ from zero beyond the 3 mm mark (or 3400 min, Figure 4B), its dynamics appear more stable than the sharper dynamics of *Actin11*. Both transcripts appeared to be under distinct control. The dynamics of *EF1β *were much slower than the dynamics of *Actin11*, which varied very rapidly in the ageing cell. However, both were maintained at an almost constant rate of net accumulation for approximately 1500 minutes (25 hours) at the very tip of the root. These results offer novel insights into the developmental analysis of gene regulation in growing plant organs.

## Discussion

### Specification of space, time and age in the study of dynamic processes

The present work focuses on the combination of two dynamic processes: expansive growth and gene expression. The analysis of both processes is influenced by the way space, time and age are considered. This stands as a pivotal point, since it shapes every experimental design and, therefore, drives conclusions. Dynamic analyses necessarily involve considering the rates of change in a biological structure (a cell, a piece of tissue or a segment of an organ). Whereas growth studies usually consider the rates of change (relative elemental growth rate), many investigations into the control of gene expression are based on the quantification of mRNA transcript levels, which are shaped by a dynamic interplay between the rates of synthesis and decay [3,5]. In mature tissues, where cells no longer move nor enlarge irreversibly, the transcriptional regulation of gene expression (for example environmental induction) is revealed by the temporal derivative of local transcript density. However, a further step is needed in order to capture gene regulation in growing structures by considering movement and expansion, which are taken into account by the input of the partial derivatives and the growth velocity field into the continuity equation [18]. The present work is, to our knowledge, the first study to apply this framework to transcript density, providing an unbiased view of the spatiotemporal regulation of gene expression (Figure 4E and 4F). In a steady growing structure, constant patterns of activity are displayed while material flows through it [31,32]. The developmental changes in net rates of gene expression were revealed by the convective and dilutive partial derivatives, two terms that are usually not considered in typical molecular studies of development.

We studied growth kinematics and gene expression under steady conditions of growth (window of stable growth rate, dark phase and constant temperature). In these conditions both the growth velocity and transcript density of housekeeping genes were assumed to be time invariant. When transcript density and/or velocity vary(ies) over time and are properly quantified at each time step, equation (1) has to be used [instead of simplified equation (3)]. Quantification of material derivative through equation (1) requires a larger experimental effort but is still straightforward (see for example [33]). Vital imaging of growth and gene expression, as well as high throughput quantitative analyses of gene transcript abundance, will greatly support such measurements in the near future, enlarging the potential uses of the method presented here.

In the present work, mean trends were established from the mean value of three independent measurements of ρ (transcript density or total RNA density) at every position. RNA extraction from very small samples is destructive and missing values prevented analysis at the individual root level. However, the advent of imaging techniques which provide accurate positioning and quantitative measurement of transcript level [11,12] will enable more detailed analyses of individual organs using the continuity equation. This could be a major breakthrough, as the analysis of single roots have revealed a steep relative elemental growth rate gradient between the meristem and the elongation zone, which is usually smoothed when pooling data from several roots [34]. Taking such gradients into account should reveal even more dynamic patterns of gene expression rates due to convective and dilutive terms. By the same token, our framework could be used to conduct a more detailed analysis at the level of small cell territories in order to characterize the contributions of different cell types along the root growth zone [8], but would require more advanced three dimensional kinematic and a more complex formalism. For simplification, the growth field of the root apex was considered here as a one dimensional vector field (the main axis) due to the slenderness of the root and the anisotropy of its growth and to the lack of shear growth (this is similar to the rod approximation in the beam theory of solid mechanics [24]). However, two or even three dimensions can be taken into account by using equations developed for appropriate coordinate systems (cylindrical, rectangular, spherical), as given by Silk [18]. More complex structures with a lower level of symmetry (for example, branching roots or apical meristem of shoots) can also be considered, using natural curvilinear coordinate system and proper tensorial formalism [35]. The transcript density would have to be characterized with the necessarily accuracy in the three dimensions. While feasible, it would require a large experimental effort.

Once the rates of change have been properly computed, the next significant challenge to reveal regulation processes is the spatial and temporal specifications of these rates. In most studies of growing organs [7,36], the property of interest is described at a given developmental stage (time) as a function of the position in the growth zone (Eulerian specification, as in Figure 2A and 2B for transcript density). This can reveal spatial mechanisms such as those related to highly diffusible or transported chemicals (for example, auxin in roots [37]). An even more insightful way to describe this dynamic process is to choose an element and track its properties as it is displaced in space and time and undergoes developmental changes (Lagrangian specification, Figure 5). These three dimensional plots are necessary in order to handle the fact that, in a growing organ, time and space are intimately but non-linearly linked. In the present study, Eulerian and Lagrangian specifications of dynamic processes highlight different aspects of developmental regulation. The Lagrangian time*space specification revealed a long-term steady accumulation of transcripts of the studied genes (*EF1β *and *Actin11*), as well as total RNA in the first 2 mm of the root tip (presumably the meristem) followed by highly dynamic regulation as the cells move away and undergo the accelerating and decelerating phases of expansion growth.

### Towards a dynamic analysis of transcriptional regulation during development

A deeper understanding of the mechanisms underlying organ growth and development requires quantitative data on three dimensional morphology and gene expression at a variety of stages and scales [13]. Recent technological advances have made it possible to analyse gene expression in individual cell types, tissues or organs through the use of specific mutants [7,8,38]. Fluorescent proteins can thus be coupled with high-resolution imaging in order to display the expression patterns of a gene at whole-organ level. For example, the expression of the vein marker gene *ATHB8 *can be used to track the earliest stage of vein development in leaves of *Arabidopsis *seedlings [39]. This approach has also been used to study tissue organization and gene expression during phloem development [40]. However, even if the analysis of consecutive snapshots reveals changes in gene expression, their spatiotemporal dynamics need to be clarified in order to avoid misinterpretation of the observed variations.

Understanding how signals through gene expression generate patterns and govern ontogenic timing is a crucial challenge in developmental biology. Coupling quantitative data with mathematical models provides some answers [41]. In the present study, real-time PCR associated with kinematic analysis of growth at high spatial resolution gave access to the dynamics of gene regulation (Figure 5A and 5B). The demonstration was conducted on two widely-studied genes considered to be housekeepers. We chose the gene coding for ACTIN11 as this protein is one of the main components of cell architecture. Actin microfilaments are involved in many processes such as cell polarity, division and elongation [42]. The use of an inhibitor of actin polymerization (latrunculin B), at a concentration sufficient to remove a considerable amount of actin, reduced the root growth rate by about 50% [43]. Using *Arabidopsis *mutants, Ringli *et al*. [44] demonstrated that the *Actin2 *gene is involved in root hair growth and Bannigan and Baskin [45] pointed out that Actin microfilaments are crucial for the tuning of cell expansion throughout the organ. In line with these findings, the expression of poplar *Actin11 *appears to be tightly controlled along the root growth zone (Figure 5A). Three pulses of up-regulation were observed, corresponding to: (i) the division zone; (ii) the accelerating part of the elongation zone; and (iii) to just before the entry into the maturation zone. The putative *Elongation factor 1-β(EF1β*) encodes a subunit of the EF-1 complex, an essential enzyme for protein synthesis in eukaryotes [46]. EF1α, another part of the same functional EF-1 complex, is accumulated in regions of high protein synthesis [47]. *EF1α *is used as a marker for meristematic activity in maize [48]. Poplar *EFIβ *was found to be up-regulated in the first two mm of the growth zone (Figure 5B). The high regulation of *Actin11 *and *EF1β *expression and their patterning along root growth zone pointed out that these genes did not display the expression stability required for their use as endogenous controls in real-time PCR.

## Conclusions

In contrast with the transcript density profiles widely used in the literature [7,8,36], the material derivative of transcript density provides key information about gene expression patterns, such as the location and the intensity of gene induction/repression. This framework appears to be very useful, offering rapid tracking of gene regulation, even in non-model species and species that are not-easily transformable. Moreover, contrary to green fluorescent protein/β-glucuronidase tracking, the framework makes it possible to analyze many genes in the same biological sample (the number is only limited by the available amount of RNA). In addition, this approach can be used: (i) to test hypotheses on when and where a transduction signal is perceived, which is a key step in rebuilding the between-gene interplays underlying growth; (ii) to unravel the temporal and spatial aspects of gene regulation between treatments affecting the time-course of cells across the growth zone, even under non-steady state situations; and, ultimately, (iii) to assess putative reference genes in the very dynamic context of growing and developing tissues. Although illustrated here for plant roots, the method is fairly generic and can be applied to most developing plant or animal organs or tissues, as long as they can be considered as a continuum [13,14].

## Methods

### Cultivation condition

Poplar cuttings (*Populus deltoïdes × nigra *cv 'Soligo') were grown in hydroponics in a controlled-environment growth cabinet (air temperature: 21°C, relative humidity: 70%, 16 h light regime, photosynthetic photon flux density: 200 μmol s^-1 ^m^-2^). The Hoagland 1/2 nutrient solution was supplemented with 0.8 mM KH_2_PO_4 _and pH was adjusted to 5.8. It was aerated by air bubbling and renewed once a week. This production of plant material was done in opaque plastic pots to avoid root exposure to light.

### Root growth

The growth rate of a root depends on its total length or its age (the time in days between its emergence and its sampling). Root growth is slow when emerging, constant for several days and then decreases (due to the competition of the lateral roots). Roots were sampled in the plateau phase and were 5-11 days old. Six roots were used to assess the velocity profile. Cuttings were sampled during the dark period and transferred for measuring velocity to a dark room where temperature was kept constant. Root growth measurements were conducted in continuous dark in order to avoid possible actinic light artefact. As poplar roots are plagiotropic, growth was assessed in a horizontal 700 mL tray filled with the same aerated nutrient solution. First, the root apex was carefully marked with graphite powder under 'safe' green light. The cutting was then fixed vertically, the base plunging in the tray so that the marked root was submerged. Time lapse photography was performed using a Canon Powershot S80 camera placed on a Leica MZ6 modular stereomicroscope, which was set vertically above the tray. A ruler was set close to the root for calibration. After a 1 h rest, a series of pictures was taken at 10 min intervals under a 'safe' green light (switched on for 1 min every 10 min). The MATLAB-based software Kineroot [49] was used to track the displacement of graphite particles over space and time and determine the velocity (the local rate of longitudinal displacement due to growth of cells along the growth zone) at a resolution of 0.25 mm.

### Transcript density

The root apices were harvested after velocity measurement. The apical centimetre, including the root cap, was cut into 1 mm-long segments under a stereomicroscope in RNA later (Ambion, Texas, USA) following the manufacturer's protocol. Total RNA was extracted using an RNeasy Micro Kit (Qiagen, CA, USA) with an additional DNAse I treatment, following the manufacturer's protocol. Total RNA concentration was determined in duplicate using a Quant-iT Ribogreen RNA Kit (Molecular Probes, Oregon, USA) and NanoDrop 1000 spectrophotometer (Thermo Scientific, Colorado, USA) following the manufacturer's protocols. RNA quality was assessed using an Experion RNA HighSens Analysis kit (Bio-Rad, CA, USA). Since total RNA content varied up to 14-fold between root segments, reverse transcription was performed on a standardized 100 ng RNA using an iScript cDNA Synthesis Kit (Bio-Rad). An Alien QRT-PCR Inhibitor Alert kit (Stratagene, CA, USA) was used to assess reverse transcription efficiency, following the manufacturer's protocol. Real-time PCR was performed in a 96-well thermocycler (MJ research PTC 2000) with a chromo4 detection system (Bio-Rad) according to the recommended cycling program (5 min 95°C, 40 cycles of 15 s at 95°C, and 1 min at 60°C) followed by the generation of a dissociation curve to check for specificity of amplification. The mix contained MESA GREEN qPCR MasterMix (Eurogentec, Liège, Beligum), 500 nM gene-specific primers and 2.5 μL cDNA (diluted 1/5) in each 15 μL reaction. *Actin11 *(CA824001 similar to At3g12110) and a putative *Elongation Factor 1-β *(BI125345 similar to At2g18110) were amplified using previously published primers [28]. Primer efficiencies were close to 100% and calculated using Opticon Monitor v3.1 software (Bio-Rad) on standard curves generated using a fourfold dilution series of all cDNA, over at least five dilution points measured in duplicate. By setting the most diluted point of the standard curve to one copy, threshold cycle values were linearly transformed into arbitrary units. Transcript abundances (a.u.) were assessed on two technical replicates. Given that all real-time procedures were conducted on standardized 100 ng RNA, transcript linear density (a.u. mm^-1^) was calculated as the transcript abundance with respect to total RNA content in each segment. For simplification, the root was considered as a homogeneous cylinder.

### Numerical methods

The system of differential equations applied here is reviewed in Silk and Erickson [22] and Silk [18]. The material derivative D (the rate of change of any property ρ in a tissue element of a growing organ) can be computed from the continuity equation:(1)

Here ρ is the transcript density (a.u. mm^-1^) or total RNA density (ng mm^-1^), v the velocity of the tissue element (mm min^-1^), x the distance from a reference point (here the root tip), and t the time (min), δρ/δt is the local partial time-derivative at position x, δρ/δx is the spatial gradient in ρ, and δv/δx is the growth velocity gradient, which measures local growth activity and has previously been termed 'relative elemental growth rate' [18] or 'growth induced strain rate' (, [24]). D is the material derivative (taking into account the movement and expansion in length of each tissue element) with (v δρ/δx) the convective component and (ρ δv/δx) the dilutive component. Contrary to most studies on the dynamics of fluids with low compressibility, this dilutive component cannot be neglected in growing tissues [32].

At any given time (t):(2)

As a reasonable first approximation (see Additional File 2: Figure S2), ρ is assumed to be steady (time invariant) and the equation is simplified:(3)

Using equation (3), we computed the material derivative D of (i) the transcript density of *Actin11 *and *EF1β *and (ii) total RNA density. Note that if the investigated property exhibits temporal variations (waves, circadian,...), the sampling method should be adequately adapted to accurately assess δρ/δt, the local partial time-derivative over time and space [equation (2)].

As the transcript density ρ was determined every mm, data were interpolated using cubic spline interpolation and setting the end second derivatives to zero (MATLAB, The MathWorks, MA, USA). The spatial derivatives of velocity (δv/δx) and transcript density (δρ/δx) were calculated using the five-point quadratic differentiation formula [50] to filter high frequency noise. Applying this formula with a 0.25 mm resolution restricted the calculation of D to the 1-9 mm range but avoided potential boundary effects.

RNA extraction from very small samples and real-time PCR are subject to experimental hazards, leading to missing values. While growth was measured on six independent roots, transcript densities ρ for every position on the root axis were measured in three out of the six roots. In addition to the calculation of mean D profiles (from the mean profiles of ρ and v), dispersion around mean D values was estimated on 1000 bootstrap replicates. The 1000 computed profiles were randomly generated by sampling with replacement of the original dataset, which consisted of three (ρ, v) pairs for every position on the root axis. Unless specified, graphs were computed using Sigmaplot software.

Under the assumption of steady state of growth (see Additional File 1: Figure S1), the growth trajectory (element position as function of time) was computed by temporally integrating the (steady) velocity. The successive positions of a particle were iteratively tracked according to x_(t+dt) _= x_(t) _+ v_(x) _dt. The initial position of the particle was set at 1 mm of the root tip in order to avoid discrepancies due to the very low velocity in the first millimetre. Under the assumption that both growth trajectory and ρ profile were constant, they were compiled to draw a Lagrangian specification of gene expression and gene regulation. Note that Lagrangian specifications can be drawn for unsteady parameters provided that the temporal variations are taken into account.

## Abbreviations

EF: elongation factor; real-time PCR: real-time polymerase chain reaction.

## Authors' contributions

RM participated in the design of the study, carried out the kinematics and molecular studies and drafted the manuscript. BM had the original idea of applying the continuity equation to the analysis of gene expression dynamics, conceived the study, participated in its design and drafted the manuscript. IH participated in the design and coordination of the study, performed the statistical analysis and drafted the manuscript. DC helped in the molecular studies and to draft the manuscript. ED helped to draft the manuscript. MBBT conceived the study, participated in its design and coordination and drafted the manuscript. All authors read and approved the final manuscript.

## Supplementary Material

Additional file 1 **Figure S1**. Data supporting the time stability of growth. (A) Velocity profiles determined by time lapse photography and Kineroot [49]. Time corresponds to the time between beginning of the dark and the sampling time. The mean velocity shown in Figure 1A is the mean ± standard error of mean of these six profiles. (B) Biplot between velocity measured during the light phase and velocity measured after 72 h continuous dark (*n *= 3, mean ± standard deviation). The relation is not statistically different from the identity function - that is, intercept = 0, slope = 1. Click here for file

Additional file 2 **Figure S2**. Data supporting the time stability of RNA profiles. (A) Profiles of *Actin11 *transcript density. Blue and red symbols correspond to the data shown in Figure 2A. Data were split into two groups according to their sampling time. The black lines correspond to complementary data - three independent roots collected after more than 72 hours of continuous dark. (B) Additional arguments for the steadiness of expression profile. The assumption of a steady profile for *Actin11 *is supported by the mining and viewing of diurnal and circadian microarray data from *Arabidopsis *and poplar http://diurnal.cgrb.oregonstate.edu. Figure S2B shows the diurnal patterns of *Actin *expression in leaves, which are the most exposed to light and presumably the most light-sensitive organ. The *Arabidopsis *ortholog (At3g12110) (dashed lines) exhibited a diurnal regulation of its expression with no circadian persistency. On the contrary, poplar *Actin11 *(continuous lines) showed a very constant expression over the diurnal cycle and no circadian rhythm. The absence of circadian control in poplar leaf supports the hypothesis of constancy of *Actin11 *expression in roots. Blue and red = 12 h light/12 h dark; black = continuous light; dashed black = continuous dark. Temperature was constant (22°C). Click here for file

Additional file 3 **Movie1**. Rotating spatio-temporal mapping - *Actin11*. Visualization of the three dimensional Figure 5A rotating from the temporal axis to the spatial axis. Click here for file

Additional file 4 **Movie2**. rotating spatio-temporal mapping - *EF1β*. Visualization of the three dimensional Figure 5B rotating from the temporal axis to the spatial axis. Click here for file

Additional file 5 **Movie3**. Rotating spatio-temporal mapping - total RNA. Visualization of the three dimensional Figure 5C rotating from the temporal axis to the spatial axis. Click here for file
