# Supplementary figures and images for: Monitoring the regulation of gene expression in a growing organ using a fluid mechanics formalism
Source: BMC Biol. 2010 Mar 4;8:18. doi: 10.1186/1741-7007-8-18 (PMC2845557; doi:10.1186/1741-7007-8-18)

## Slide 1
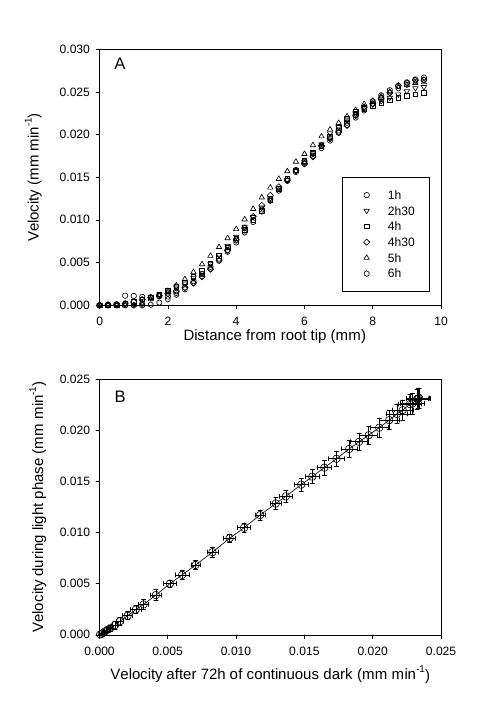

Supplement: Additional file 1 — Figure S1. Data supporting the time stability of growth. (A) Velocity profiles determined by time lapse photography and Kineroot [49]. Time corresponds to the time between beginning of the dark and the sampling time. The mean velocity shown in Figure 1A is the mean ± standard error of mean of these six profiles. (B) Biplot between velocity measured during the light phase and velocity measured after 72 h continuous dark (n = 3, mean ± standard deviation). The relation is not statistically different from the identity function - that is, intercept = 0, slope = 1. [file 1741-7007-8-18-S1.PPT]

## Slide 1
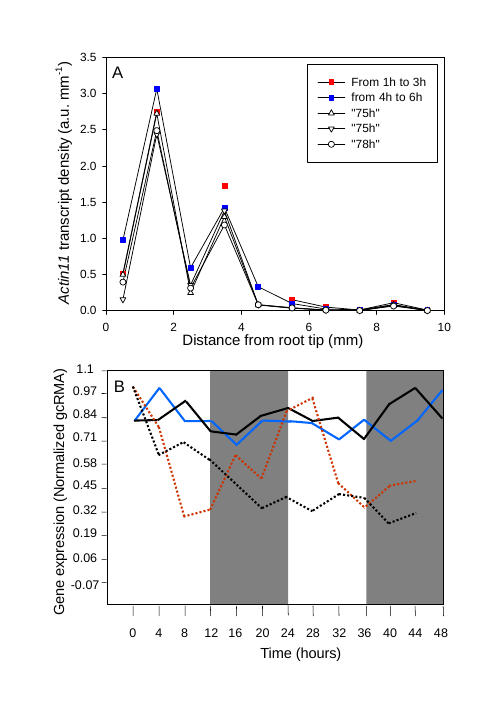

A
1.1
0.97
0.84
0.71
0.58
0.45
0.32
0.19
0.06
-0.07
Gene expression (Normalized gcRMA)
B
0
4
8
12
16
20
24
28
32
36
40
44
48
Time (hours)

Supplement: Additional file 2 — Figure S2. Data supporting the time stability of RNA profiles. (A) Profiles of Actin11 transcript density. Blue and red symbols correspond to the data shown in Figure 2A. Data were split into two groups according to their sampling time. The black lines correspond to complementary data - three independent roots collected after more than 72 hours of continuous dark. (B) Additional arguments for the steadiness of expression profile. The assumption of a steady profile for Actin11 is supported by the mining and viewing of diurnal and circadian microarray data from Arabidopsis and poplar http://diurnal.cgrb.oregonstate.edu. Figure S2B shows the diurnal patterns of Actin expression in leaves, which are the most exposed to light and presumably the most light-sensitive organ. The Arabidopsis ortholog (At3g12110) (dashed lines) exhibited a diurnal regulation of its expression with no circadian persistency. On the contrary, poplar Actin11 (continuous lines) showed a very constant expression over the diurnal cycle and no circadian rhythm. The absence of circadian control in poplar leaf supports the hypothesis of constancy of Actin11 expression in roots. Blue and red = 12 h light/12 h dark; black = continuous light; dashed black = continuous dark. Temperature was constant (22°C). [file 1741-7007-8-18-S2.PPT]
